# Selected Vitamins and Essential Elements in Meat from Semi-Domesticated Reindeer (*Rangifer tarandus tarandus* L.) in Mid- and Northern Norway: Geographical Variations and Effect of Animal Population Density

**DOI:** 10.3390/nu4070724

**Published:** 2012-07-10

**Authors:** Ammar Ali Hassan, Torkjel M. Sandanger, Magritt Brustad

**Affiliations:** 1 Centre for Sami Health Research, Department of Community Medicine, Faculty of Health Sciences, University of Tromsø, Tromsø N-9037, Norway; Email: torkjel.sandanger@uit.no or tsa@nilu.no (T.M.S.); magritt.brustad@uit.no (M.B.); 2 Norwegian Institute for Air Research (NILU), Fram Centre, Tromsø N-9296, Norway

**Keywords:** Sami, reindeer, meat, vitamins, essential elements, geography, animal population density

## Abstract

Meat samples (*n* = 100) were collected from semi-domesticated reindeer originating from 10 grazing districts in Norway. We aimed at studying concentrations, correlations, geographical variations and the effect of animal population density on vitamins A, B3, B7, B12 and E, and calcium, iron, zinc, selenium, chromium and cobalt. Mean concentrations of vitamins A, B3, B7; B12 and E were <5 µg, 6.6 mg, <0.5 µg, 4.7 µg and 0.5 mg/100 g wet weight, respectively. Concentrations of calcium, iron, zinc, selenium, chromium and cobalt were 4.7 mg, 2.8 mg, 6.4 mg, 19.4 µg, 1.7 µg and 0.5 µg/100 g wet weight, respectively. Vitamin E and selenium were the nutrients that exhibited the largest geographical variations (*p* < 0.05), although no geographical gradient was observed for any of the studied nutrients. Age had a significant effect on zinc and selenium concentrations. Iron was significantly positive correlated with calcium (*r* = 0.3416, *p* < 0.01) and vitamin B12 with zinc (*r* = 0.35, *p* < 0.05). Reindeer from districts with low animal population density had significantly higher selenium concentration than those from districts with medium and high population densities (*p* < 0.01). Reindeer meat contained higher vitamin B12, iron, zinc and selenium concentrations when compared to Norwegian beef, lamb, mutton, pork and chicken meat.

## 1. Introduction

Red meat consumption has previously been stated as a risk factor for cardiovascular diseases (CVD) and colon cancer due to the fatty acids composition [1]. In contrast to this, reindeer meat has a desirable fatty acid profile. Moreover, the limited data on reindeer meat has revealed higher nutrient contents (e.g., vitamin B12 and iron) when compared to other red and white meat types [2,3,4]. Reindeer as ruminant animals preserve the ability of synthesizing a large amount of vitamin B12 (when cobalt is present) as a result of rumen microbial activity. The vitamin B12 is then stored in liver and meat, and represents three to five times the amount found in meat from mono-gastric animals such as pigs and poultry [5,6,7,8].

Reindeer husbandry in Norway is restricted by law to the Sami indigenous people and is based on a free range herding system all the year around [9]. The reindeer move between different grazing districts during the year due to the varying natural conditions and nutritional demands [10]. These movements are man-controlled as well as season-dependent (summer *vs*. winter). The natural conditions such as geology, degrees of snow depth and formation of ice crusts, length of summer season, presence and intensity of parasites especially warble flies (*Hypoderma tarandi*) and possible replacement of vegetation system with species that are not favoured by reindeer vary across geography [11,12,13,14]. Furthermore, the varying density of reindeer population and grazing areas available for reindeer pasture (capacity) may as well result in different degrees of supplement-feed (e.g., pellet concentrates and hay) practice, as districts suffering overgrazing are more likely to rely on such practice more often than ones less affected. These variations may lead to different pasture qualities and varying degrees of pasture utilities by reindeer among grazing districts. Thus, variation in nutrient concentrations in meat from reindeer are expected between districts as indicated in our previous study [2].

The main purpose of this work was to increase knowledge about nutrients in reindeer meat by studying geographical variations and the effect of animal population density on selected vitamins and essential elements in meat from semi-domesticated reindeer originating from mid- and northern Norway.

## 2. Experimental Section

### 2.1. Geographical Area

**Figure 1:**
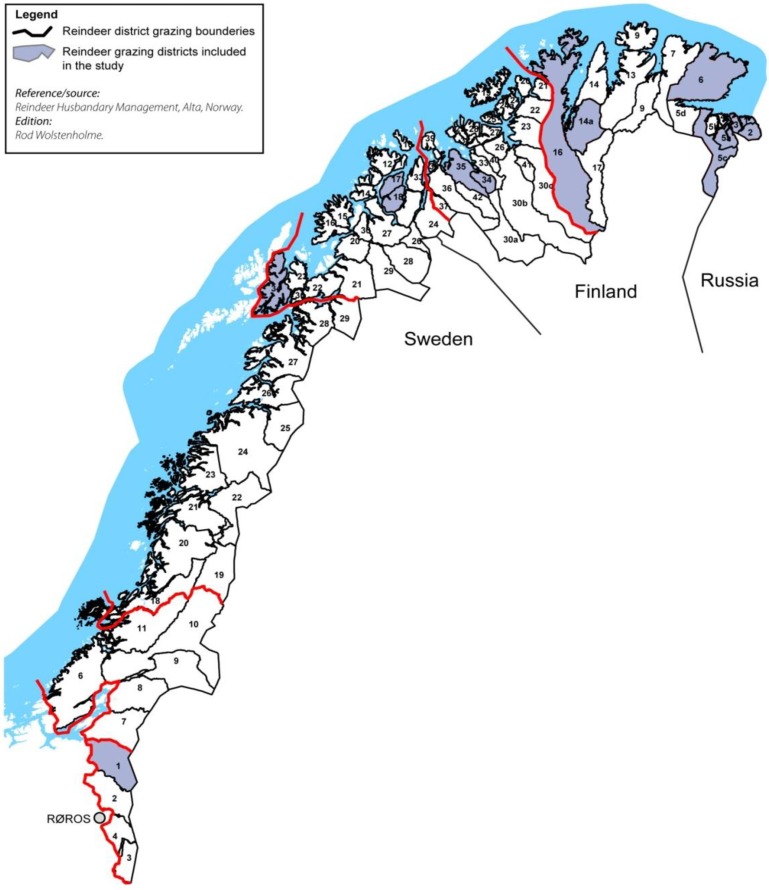
Map of the study area.

The meat samples (*n* = 100) were collected from ten grazing districts (equal number, *n* = 10) from four different counties distributed as follows: Finnmark County (7 districts), Troms County (one district), Nordland County (one district) and Sør-Trøndelag (one district). The selection of the ten districts was based on obtaining broad geographical and animal population density ranges (Figure 1). The selection of seven districts from Finnmark which is the biggest and the northernmost Norwegian county was based on the fact that this county has the largest number of semi-domesticated reindeer and 50% of the total number of the reindeer grazing districts in Norway.

### 2.2. Sample Collection and Preparation

Meat samples (*n* = 100) from the dorsal neck region (pure muscles) were collected from reindeer in ten different grazing districts located in the north and middle of Norway in the period from October to December 2008 and September to December 2009. Random collections were not carried out. However, references to randomization are not needed since the total population was analysed based on the fact that reindeer from different herds within the same district were transported together to the slaughterhouse and slaughtered on the same day. Thus, reindeer from almost all herds within the same district were represented in the sampling process. For comparison reasons we aimed at collecting samples from young animals (1.5 years old). However, due to limited availability of 1.5 years old in some districts (*n* = 4), calves (approximately10 months old) and old animals (>2 years old) with the respective proportions of 12% and 11% were selected. Thus, 77% of the total samples consisted of young animals. The age of the reindeer was obtained directly from the tags attached to the animals’ carcasses when they passed the weighing post in the slaughterhouses. There were 52 males and 48 females of the 100 selected reindeer. All samples (composed of all dorsal neck muscles) were collected directly after the slaughter/dressing process with carcass weighing in pre-marked plastic bags prior to further division into dedicated glass containers. Glass containers with samples for vitamins analyses were covered with aluminium foil to prevent them from being exposed to light. The samples were kept cool in a cooling box (approximately 4 °C) immediately after collection and division, and then moved the same day to a −20 °C freezer until they were freighted frozen to the laboratory for analysis. All animals from which samples were collected were healthy ones, *i.e.*, passed the ante- and post mortem inspection.

Due to the high cost of vitamin analysis, pooled samples of raw meat (with aliquots of muscles) from each of the five animals originating from the same district (two pooled samples per district) were prepared.

### 2.3. Chemical Analyses

#### 2.3.1. Vitamins

The analyses of vitamins were done by GBA-Food, Hamburg, Germany according to methods of German Food Act LMBG § 35, LFGB § 64 and standard methods of Association of Official Analytical Chemists (AOAC) [15,16]. The laboratory is accredited for the methods used in the analyses according to Staatliche Akkreditierungsstelle Hannover, AKS-P-20213-EU. The vitamins A, B3 and E concentrations refer to retinol, niacin and α-tocopherol, respectively. Vitamins A, B3, B12 and E were analysed using normative reference § 35 LMBG/DIN EN 12823, AOAC 944.13, AOAC 952.20 and § 64 LMBG/DIN EN12822, respectively. For vitamin B7, an internal method was employed using hot acid (HCl) hydrolysis in the extraction, and microbiological/turbidimetric method in detection and external standard in quantification. Measurement of uncertainty for vitamins analyses were given as extensive uncertainty measurement according to (Guide to the expression of uncertainty in measurement, ISO, Geneva, Switzerland) estimated by a covering factor of 2 (95% confidence interval). The selection of the studied vitamins and essential elements was based on data from a previous study by the same authors [2].

#### 2.3.2. Essential Elements

The samples were separately decomposed using a microwave oven (Ethos Plus, Milestone Inc., Shelton, CT, USA) and concentrated supra-pure HNO_3_ (5 mL) and H_2_O_2_ (3 mL) were added to the sample (0.6–0.7 g) before undergoing the microwave oven treatment. Then, the following temperature regimes were subsequently used in the microwave oven: 20–50 °C (5 min), 50–100 °C (10 min), 100–180 °C (5 min) and 180 °C (15 min). After cooling down the heated decomposed sample, the solution was diluted to 50 mL. The sample solution was analysed using an inductively coupled plasma high resolution mass spectrometer (ICP-HRMS), Bremen, Germany. All standards and calibration solutions contained 1 ppb Rhenium (Re) as the internal standard, together with 1% nitric acid (HNO_3_). The calibration curve was verified by standard quality control (QC) sample (Spex Standard, Ultra Scientific, North Kingston, RI, USA) in compliance with ANSI/NCSLZ-540-1 and ISO 90001. The QC material SRM-1566a (Oyster tissue) was obtained from the National Institute of Standards and Technology (NIST), Maryland, USA. The resolutions used for essential elements were low (10) for (Zn), middle (20) for (Ca, Fe), and high (30) for (Se). Lens-adjustment was optimized daily to ensure maximum intensity and top separation. The analyses were done by the NILU (Norwegian Institute for Air Research) Laboratory, Kjeller, Norway. The laboratory is accredited for the methods used in the analyses according to NS-EN ISO/IEC 17025, No. TEST008. The limits of detection (LODs) for the studied essential elements were three times standard deviation (SD) of the laboratory blanks, whereas the limits of quantifications (LOQs) were 10 times the SD of the blanks, decomposed simultaneously with the meat samples.

### 2.4. Statistical Procedures

STATA/SE 12.0 for Windows (STATA Corp. College Station, TX, USA) was used for statistical analyses. Laboratory results for vitamins and essential elements below the limit of detection (LOD) were given a numerical value of zero. A standardized residuals test was conducted in order to check for possible outliers in essential elements concentrations (observations that were more than three standard deviations from the mean). All outliers were removed (*n* = 7, details in results and discussion parts).

Analysis of variance and covariance (ANOVA), with the specific essential element as a dependent variable and districts/animal density, age and sex as independent variables, was used to test for the effect of animal population density on essential element concentrations. Animal population density was categorized, on the basis of the 10 districts included in the study, into three groups; low (0.8–1.9, *n* = 5 districts), medium (3–5.3, *n* = 4 districts) and high (6–13.7, *n* = 1 district) animals/km^2^. The animal population density (animal/km^2^) for each reindeer grazing district was calculated according to available data and based on the following formula:

Animal population density = Number of reindeer in a specific district/total area of the district

The district areas were given in square kilometres (km^2^).

For vitamins, the final model consisted of the specific vitamin as dependent variable and animal density as an independent variable. Due to presence of some pooled samples (for vitamins analyses) with mixed age and sex groups from four districts (*n* = 8 pooled samples), additional statistical analyses were done to look into the effect of age and sex on vitamin concentrations using pooled samples from the homogenous age and sex groups (more details on statistical analysis for vitamins in discussion part). The relationship between vitamins B3 and B12, and animal population density was U-formed (non-linear), therefore we log transformed the animal density variable and performed a regression analysis on vitamins B3 and B12.

Bonferroni multiple comparison tests were used to test for significant differences in concentrations of vitamins and essential elements among the grazing districts using the specific vitamin/essential element as a response (dependent) and the districts as a factor variable. Welch test was used whenever homogeneity of variance was violated. The overall vitamin concentrations were given as grand mean (mean of the different pooled samples, *n* = 20 pooled samples composed of 100 individual samples, *i.e.*, five individual samples in each pooled sample originating from the same grazing district). Pairwise correlation tests with Bonferroni-adjusted significance level were used to test for possible correlations within/between vitamins and essential elements. The level of statistical significance was set at *p* < 0.05 for all the statistical analyses.

## 3. Results

Results of essential element concentrations were presented as mean per 100 g raw meat, standard deviation (SD) and Minimum-Maximum. For vitamin concentrations in samples from the different districts, the results were presented as concentrations of the two pooled samples from each district. Furthermore, the overall vitamin concentrations were given as a grand mean (mean of the 20 pooled samples).

### 3.1. Vitamins

Vitamin A was below the limit of detection (<5 µg/100 g) in all pooled samples, except for one from Essand (Røros) with a concentration slightly above the limit of detection (5.7 µg/100 g), whereas vitamins B3, B12 and E were above the limits of detection (LOD) in all pooled samples. Vitamin B7 was detected (>0.5 mg/100 g) in five pooled samples from five districts with a minimum concentration equal to the LOD and a maximum one of 0.3 µg above the LOD. The overall vitamin concentrations are presented in Table 1. 

**Table 1:** The overall grand mean concentrations of the studied vitamins per 100 g raw meat (*n* = 20 pooled samples composed of 100 individual meat samples.

Vitamin	Mean	SD	Minimum	Maximum	Percentage (%) of pooled samples over LOD	LOD
Vitamin A (µg)	<5		<5	5.7	5%	5
Vitamin B3 (mg)	6.6	0.8	4.7	7.7	100%	0.2
Vitamin B7 (µg)	<0.5	0.1	<0.5	0.8	25%	0.5
Vitamin B12 (µg)	4.7	1.7	1.7	8.8	100%	0.1
Vitamin E (mg)	0.5	0.2	0.3	0.8	100%	0.002

SD = Standard deviation; LOD = Limit of detection. Note: Pooled samples (*n* = 20 pooled samples from 100 individual samples). Standard deviation (SD) is not given for vitamin A as only one pooled sample (*n* = 5 individual samples) had a concentration above the limit of detection.

No further statistical analyses were done on vitamin A and B7 due to the low percentage of samples above the LOD. A significant positive correlation was observed between vitamin B12 and Zn (*r* = 0.35, *p* < 0.05).

### 3.2. Essential Elements

The studied essential elements were detected in all meat samples (100%), except for cobalt (Co) in which two samples had concentrations below the limit of detection (<0.12 µg/100 g). The overall mean concentrations of essential elements are presented in Table 2. Concentrations of selenium (Se), cobalt (Co), zinc (Zn) and chromium (Cr) were significantly different (*p* < 0.05) between some of the districts. Sex had no significant effect for any of the essential elements, whereas age had a significant effect on Zn concentrations (*F* = 3.26, *p* < 0.05).

**Table 2:** Overall mean concentrations of the selected essential elements per 100 g raw meat.

Element	*n*	Mean	SD	Minimum	Maximum	LOD	LOQ
Ca (mg)	99	4.7	1.3	1.9	10.8	0.07	0.24
Fe (mg)	99	2.8	0.7	1.5	4.6	0.01	0.03
Zn (mg)	100	6.4	1.6	2.6	10.7	0.01	0.03
Se (µg)	98	19.4	10.1	7.1	51.5	1.55	5.15
Cr (µg)	99	1.7	2.9	0.1	16.2	0.10	3.45
Co (µg)	98	0.5	0.3	<0.1	1.7	0.09	0.31

SD = Standard deviation; LOD = Limit of detection; LOQ = Limit of quantification. Note: Number of samples (*n*) less than 100 for some essential elements were due to exclusion of outliers (*n* = 7) from statistical analyses.

Essential element concentrations in some samples (*n* = 7) were outliers and have been removed from the statistical analyses. The detected outliers were: One animal (1.5 years) from Karasjok West with a calcium concentration of 56.9 mg/100 g; one animal (calf) from Pasvik with a Fe concentration of 60.8 mg/100 g; two animals (1.5 years) from Eastern Sør-Varanger with Se concentrations of 68.1 and 68.4 µg/100 g; one animal (1.5 years) from Karasjok West with a Cr concentration of 82.4 µg/100 g; two animals (1.5 years) from Spierttagáisá with Co concentrations of 21.3 and 22.6 µg/100 g.

Calcium concentrations were in the range of 3.8–5.4 mg/100 g, whereas iron concentrations were in the range of 2.2–3.5 mg/100 g. The interaction between the districts and age in zinc and selenium models was significant (*F* = 2.59, *p* < 0.05 and *F* = 6.47, *p* < 0.01, respectively), therefore stratified analyses have been provided in Table 3 and Table 4.

**Table 3:** Age stratified Zn Concentration (mg/100 g wet weight (ww)) in reindeer meat from districts with mixed age groups.

District	Mean ± SD (Minimum-Maximum) Zn Concentration			
	Calves	*n*	Young and Older Animals	*n*
Varanger Peninsula	5.5 ± 1 (4.4–6.5)	3	8.4 ± 1.5 (6.8–10.1)	7
Eastern Sør-Varanger	-- ^a^	1	6.9 ± 1.7 (4.7–10.4)	9
Pasvik/Sør-Varanger	4.2 ± 0.8 (2.8–5.1)	7	4.4 ± 2.3 (2.6–7)	3
Kanstadfjord	-- ^b^	1	6.8 ± 0.5 (6.3–7.7)	9

^a^ One calf with Zn concentration of 5.1 mg/100 g; ^b^ One calf with Zn concentration of 5.7 mg/100 g.

**Table 4:** Age stratified Se Concentration (µg/100 g ww) in reindeer meat from districts with mixed age groups.

District	Mean ± SD (Minimum–Maximum) Se Concentration			
	Calves and older animals	*n*	Young animals	*n*
Varanger Peninsula	16.9 ± 5 (10.7–22.3)	7	20.5 ± 4.1 (17.1–24.9)	3
Eastern Sør-Varanger	43.4 ± 7.2 (37.9–51.5)	3	44.4 ± 4.5 (40.1–51.5)	5
Pasvik/Sør-Varanger	30.2 ± 5.9 (24.6–43.4)	8	17.2 ± 3.4 (14.7–19.6)	2
Kanstadfjord	25.5 ± 4.7 (17.1–28.9)	5	28.4 ± 2.2 (25.6–30.7)	5

Young animals (1.5 years) and older ones (>2 years) demonstrated no significant difference in Zn concentration between districts (Mean = 6.6 and 7.1 for young and older animals, respectively), while calves showed a lower mean Zn concentration (4.72, *p* < 0.01). Moreover, there was no significant difference in Se concentrations between calves and older animals (25.6 and 25.8 µg/100 g, respectively), whereas young animals (1.5 years) had a significantly lower Se concentration (16.6 µg/100 g, *p* < 0.05).

Chromium and cobalt were the least abundant essential elements in reindeer meat and had mean concentrations of 1.7 and 0.5 µg/100 g, respectively. Significant positive correlations were revealed only between calcium and iron (*r* = 0.34, *p* < 0.05), and zinc and vitamin B12 (*r* = 0.35, *p* < 0.5). 

### 3.3. Geographical Variations

Geographical variations were revealed in concentrations of vitamins B3, B12, E, zinc, selenium, cobalt and chromium, with vitamin E and selenium demonstrating the largest geographical variations. None of the studied nutrients demonstrated a geographical gradient as could be seen in Table 5 and Table 6. 

The district Essand (Røros) was distinguished by being the only district from which vitamin A concentration was above the LOD (one pooled sample). Additionally, Eastern Sør-Varanger, Karasjok West, Ábborašša, Tromsdalen and Essand were the districts that had vitamin B7 concentrations (from one pooled sample each) above the LOD. The district Tromsdalen was distinguished by its high vitamins B3 and E as well as Cr, whereas Eastern Sør-Varanger, Pasvik and Kanstadfjord by their high Se concentrations.

Furthermore, the districts Eastern Sør-Varanger, Pasvik, Fávrrosorda and Spierttagáisá were distinguished by their high Co concentrations.

**Table 5:** Concentration of vitamins per 100 g reindeer meat (ww) from the different districts.

District	Pooled sample 1–Pooled sample 2 (*n* = 20 pooled samples)			
	B3 (mg)	B7 (µg)	B12 (µg)	E (mg)
Eastern Sør-Varanger	5.1–6.6	<0.5–0.5	5–5.7	0.5–0.6
Pasvik	7.2–7.6	<0.5–<0.5	3.3–5	0.5–0.5
Varanger Peninsula	4.7–6.4	<0.5–<0.5	3.2–8.8	0.4–0.8
Spierttagáisá	5.8–6.7	<0.5–<0.5	3.9–4.1	0.5–0.7
Karasjok West	6.7–7.1	<0.5–0.7	2.7–7.8	0.3–0.3
Ábborašša	6.7–7.2	<0.5–0.6	4.7–5	0.4–0.5
Fávrrosorda	5.9–7	<0.5–<0.5	5.9–6.1	0.4–0.5
Tromsdalen	7.5–7.7	<0.5–0.5	1.7–2.1	0.8–0.8
Kanstadfjord	6.5–7.4	<0.5–<0.5	3.9–4.5	0.6–0.7
Essand (Røros)	6.1–6.6	<0.5–0.8	5–5.5	0.3–0.3

Note: Pooled sample: samples from 5 individual animals originating from the same district;Vitamin A concentrations were below the limit of detection (<5 µg) in samples from all districts; except for the district Essand (Røros) in which one pooled sample was within detection limit (5.7 µg/100 g).

**Table 6:** Concentration of the selected essential elements per 100 g (ww) across grazing districts.

District	Mean ± SD					
	(Minimum–Maximum) *n* = 10 samples per district					
	Ca (mg)	Fe (mg)	Zn (mg)	Se (µg)	Cr (µg)	Co (µg)
Eastern Sør-Varanger	4.6 ± 0.6	3.1 ± 0.6	6.7 ± 1.7	44 ± 5.2	0.6 ± 0.7	1 ± 0.4
	(4.1–5.9)	(2.2–4.2)	(4.7–10.4)	(37.9–51.5)	(0.2–2.4)	(0.6–1.7)
Pasvik/Sør-Varanger	4.9 ± 0.9	3.5 ± 0.7	4.3 ± 1.3	27.6 ± 7.7	0.9 ± 0.4	0.6 ± 0.3
	(4.3–6.5)	(2.3–4.6)	(2.6–7)	(14.7–43.4)	(0.4–1.6)	(0.3–1.1)
Varanger Peninsula	5.1 ± 1.2	2.8 ± 0.7	7.6 ± 1.9	17.9 ± 4.9	0.4 ± 0.3	0.4 ± 0.2
	(3.8–7.5)	(2–4.3)	(4.4–10.1)	(10.7–24.9)	(0.1–1.1)	(0.1–0.9)
Spierttagáisá	4.9 ± 1.1	2.2 ± 0.5	5.7 ± 0.9	12.1 ± 2.5	3.1 ± 4.5	0.7 ± 0.2
	(3.9–7.4)	(1.7–3.3)	(4.8–7.9)	(9.3–17.6)	(0.1–12.5)	(0.5–1.1)
Karasjok West	4.7 ± 1.3	2.9 ± 0.6	6.9 ± 1.5	13.4 ± 3.3	1.2 ± 1.3	0.4 ± 0.2
	(3.6–7.9)	(1.8–3.9)	(4.3–9.4)	(7.6–18.4)	(0.3–4.4)	(0.1–0.9)
Ábborašša	4.9 ± 0.8	2.8 ± 0.6	6.4 ± 1.1	11.7 ± 1.4	1.7 ± 1.9	0.6 ± 0.2
	(3.6–7.3)	(2–4.1)	(4.5–7.6)	(8.1–12.9)	(0.2–5.4)	(0.3–0.9)
Fávrrosorda	3.8 ± 1.7	2.6 ± 0.9	7.1 ± 2.3	12.9 ± 2.8	2.1 ± 3.4	0.7 ± 0.4
	(1.9–8.3)	(1.5–4)	(4–10.7)	(9.7–17.7)	(0.3–11.6)	(0.2–1.3)
Tromsdalen/Andersdalen-Stormheimen	4.6 ± 1.2	2.9 ± 0.8	5.4 ± 0.7	12.9 ± 2.8	5.1 ± 5.8	0.3 ± 0.3
	(3.3–7.3)	(1.7–4.2)	(4.4–6.2)	(7.1–16.5)	(0.3–16.2)	(<0.1–0.7)
Kanstadfjord/Westren Hinnøy	5.4 ± 2.2	2.8 ± 0.5	6.7 ± 0.6	26.9 ± 3.8	1.2 ± 0.9	0.3 ± 0.1
	(3.8–10.8)	(2.1–3.6)	(5.7–7.7)	(17.1–30.7)	(0.2–3.2)	(0.2–0.4)
Essand/Røros	3.6 ± 0.4	2.8 ± 0.3	7.2 ± 0.9	18.8 ± 4.7	0.3 ± 0.2	0.4 ± 0.2
	(2.9–4.4)	(2.5–3.5)	(5.1–8.6)	(8.1–23.4)	(0.1–0.7)	(0.1–0.6)

Note: Number of samples (*n*) less than 10 per district (due to exclusion of outliers) for some essential elements: Eastern Sør-Varanger: *n* = 8 for Se; Karasjok West: *n* = 9 for Ca and Cr; Pasvik: *n* = 9 for Fe; Spierttagáisá: *n* = 8 for Co.

### 3.4. Animal Population Density

No significant effect for animal population density on concentrations of the studied nutrients could be observed, except for that on selenium concentration. Reindeer originating from districts with low animal population density (0.8–1.9 animals/km^2^) had on average 12.4 µg/100 g higher Se (*p* < 0.01) than those originating from districts with medium (3–5.3 animals/km^2^) and high (6–13.7 animals/km^2^) animal population densities.

## 4. Discussion

Reindeer meat contained higher vitamin B12, Fe, Zn and Se concentrations when compared to Norwegian beef, lamb, mutton, pork and chicken meat [4]. The geographical differences revealed in this study were not large and will most likely have no impact for consumers. Vitamin E and Selenium demonstrated relatively large geographical variations. Calves had a significant lower Zn concentration than young and older animals, whereas young animals had a significant lower Se concentration than calves and older animals. Positive correlations were revealed between iron and calcium, and vitamin B12 and zinc. Animals originating from districts with low animal population density had on average higher selenium concentration than those from districts with medium and high population densities.

### 4.1. Concentrations and Geographical Variations

Reindeer meat contained a concentration of vitamin B12 that is nearly four, five, nine and twelve times higher than those of lamb meat, beef, pork and chicken, respectively. Iron concentration in reindeer meat was two times higher than that of lamb meat and beef, and four times higher than that of pork and chicken. Furthermore, Zinc concentration was two times higher than that of beef, three times higher than that of lamb and pork, and five times higher than that of chicken, whereas selenium concentration was two times higher than that of pork and chicken, seven times than that of lamb and five times than that of beef [4].

Vitamin A was detected in only one pooled sample originating from Essand/Røros. Vitamin E and selenium were the nutrients that demonstrated the largest geographical variations, whereas no geographical differences were found for vitamin B7, calcium and iron concentrations. Calves (10 months) had a significant lower Zn concentration than young (1.5 years) and older animals (>2 years), whereas young animals had a significantly lower Se concentration than calves and older animals. Iron was positively correlated with calcium, and vitamin B12 was positively correlated with zinc. Districts with medium and high animal population density (3–5.3 and 6–13.7 animals/km^2^, respectively) had an average 12.4 µg/100 g raw meat lower selenium than those with low population density (0.8–1.9 animals/km^2^).

Concentration of vitamin A detected from the pooled sample originating from Essand (Røros) in this study (5.7 µg/100 g) was comparable to that reported from reindeer meat in Finland, two times higher than that from Sweden and four times lower than those previously reported from Norway [2,17,18,19]. Furthermore, vitamin A concentration reported from Arctic Canadian caribou (93.5 µg/100 g ww) was much higher than the concentration detected in the present study [20].

The concentration of vitamin B3 detected in this study (6.6 mg/100 g) was comparable to that found in US caribou, slightly higher than that previously reported from Norway (4.3 mg/100 g) and slightly lower than that reported from Finland (8.6 mg/100 g) and from Canadian caribou, 10.9 mg/100 g [2,18,21,22]. No data on vitamin B7 in meat from reindeer or caribou were available for comparison other than that of 1.2 µg/100 g from Norway which was two times higher than the value of 0.6 µg/100 g detected in the present study [2]. Vitamin B12 concentration of 4.7 µg/100 g detected in the present study was comparable to that previously reported from Norway (3.3 µg/100 g) and slightly lower that of 6.31 µg/100 g reported from US caribou [2,21]. Additionally, vitamin B12 concentration in reindeer meat was found to be higher when compared to concentrations in meat from other ruminant animals (e.g., mutton and beef) [2,3,4]. This could be due to reindeer feeding on lichens as lichens have previously been found to improve microbial activity in reindeers’ rumens [23].

Concentration of vitamin E (α-tocopherol) detected in the present study (0.5 mg/100 g) was comparable to that previously reported in reindeer meat from Norway and Sweden, lower than that of 0.84 mg/100 g reported from Finland and higher than that of 0.15 mg/100 g reported from Arctic Canada [2,18,19,20,24].

Calcium concentration of 4.7 mg/100 g detected in this study was comparable to that reported from Norway, Finland, and Arctic Canada [2,18,25]. However, Ca concentration of 17 mg/100 g previously reported from US caribou was nearly four times higher than the one detected in the present study [21].

Iron concentration in the present study was comparable to that of 3.3 and 3 mg/100 g formerly reported from Norway and Finland, respectively [2,18]. However, Fe concentration from this study was nearly 50% lower than that reported from Russian reindeer, Arctic Canadian and US caribou [21,25,26]. The highest Fe concentration of 4.6 mg/100 g measured in this study agreed well with that newly reported from a Norwegian study on reindeer meat by Triumf and colleagues [24]. Zinc concentration detected in the present study was in agreement with that previously reported from Norway and nearly two times higher than values reported from Greenlandic reindeer, Arctic Canadian and US caribou [2,21,25,27].

Selenium concentration in the present study was characterized by a wide range (7.1–51.5 µg/100 g). This was due to geographical variation in Se concentration which has also been demonstrated in our previous study on meat, liver, tallow and bone marrow from reindeer [2]. Se concentration detected in this study was comparable to that reported from Finland (24 µg/100 g), twice that of 10.2 µg/100 g reported from US caribou and much higher than that of 3 µg/100 g reported from a previous Norwegian study [2,17,18,21]. Furthermore, Se concentrations from Greenlandic reindeer ranged from 0.3 to 2.52 µg/100 g while Canadian Arctic caribou revealed much lower concentration (0.01 µg/100 g) [25,27]. No data were available for comparison regarding Cr and Co concentrations in meat from reindeer and caribou. However, Sivertsen and colleagues have reported Cr concentration of 2 µg/100 g and Co concentrations ranged from 7 to 11 µg/100 g from reindeer liver in Norway [28]. Similar Co concentrations as detected by Sivertsen *et al*. and a higher Cr (8 µg/100 g) have been detected from Russian reindeer liver [29].

Reindeer meat contained higher vitamin B12, Fe, Zn and Se concentrations when compared to Norwegian beef, lamb, mutton, pork and chicken meat [4]. Carcass cuts had been taken into consideration when the previously mentioned comparison was done. This was conducted either by using the same carcass cut (e.g., neck cutlets) or using cuts that are anatomically relevant to neck muscles (e.g., saddle muscles). The geographical differences for nutrient concentrations revealed in the present study were generally not large and will most likely have no impact for consumers. The evaluation of the vitamin and essential element concentrations in terms of high or low should be looked at in the light of the recommended dietary intake/allowance (RDI/RDA) values for each vitamin or essential element as set by the Nordic Council of Ministers/the US National Research Council [30,31].

The wide Se concentration range (µg/100 g raw reindeer meat) in the present study contributes to 18%–100% and 14%–100% of the RDI for adult women and men, respectively. Furthermore, the detected Zn range contributes to 38%–157% and 29%–122% for women and men, respectively. The detected vitamin B12 range of 1.7–8.8 µg/100 g raw reindeer meat contributes to 85%–440% of the RDI for both women and men. There are no established RDI/RDA for vitamin B7, cobalt and chromium. However a US recommendation for vitamin B7 of 30 µg per day was set as an adequate intake (AI) for adults [32]. Furthermore, chromium concentrations of 25 and 35 µg/day have been reported as adequate intakes (AI) for young women and men, respectively [33]. 

Vitamin B12 synthesis in ruminant animals (e.g., reindeer) depends on the presence of cobalt [34]. Hence, we were expecting vitamin B12 to correlate positively with cobalt, but such correlation was not observed in the present study.

### 4.2. Animal Population Density

Animals originating from grazing districts with low animal population density had on average higher Se concentrations than those from districts with higher animal population densities. This could be due to the fact that animals from grazing districts with high animal population density and limited lichens availability are more likely to experience competition for lichen sources. The lichens have previously been found to contain higher selenium when compared to other plant groups [35].

### 4.3. Pooled Vitamin Samples

Due to the presence of some pooled vitamin samples with mixed age groups (*n* = 8 pooled samples) from some districts (*n* = 4 districts), additional statistical analyses were performed in order to see whether age had an effect on vitamin concentrations or not before we could join all data together prior to the statistical analyses. This was done by dividing data into two sets; one with districts (*n* = 6 districts) that had homogenized age group (*n* = 60 animals) and the other with those (*n* = 4 districts) with mixed age group (*n* = 40 animals). We carried out the same statistical analyses as described in the statistical analysis part on both data sets and no effect for age on vitamin concentrations could be observed (results not presented). However, reindeer calves had previously been reported to have higher (7%–10%) vitamin concentrations than those of adult reindeer [17,36]. Advantages and disadvantages of pooled samples were discussed elsewhere [2]. The ideal situation in case of pooled samples is that samples need to originate from the same districts and consist of homogenized age and sex groups. However, looking into differences in vitamin concentrations within districts would not be possible in such a case as concentrations of pooled samples were based on the mean of the individual samples from which the pooled sample consisted (*i.e.*, not obtained from individual concentrations separately).

### 4.4. Limitations of the Study

The statistical comparisons carried out on geographical variations and effect of animal population density were not based on similar measured water content (moisture %) or dry weight (dw) based concentrations. The concentrations of the studied vitamins and essential elements were based on direct wet weight (ww). No data were available on water content and dry weight based concentrations. Thus, the statistical comparisons are flawed since they were conducted on a wet weight basis and the differences observed may only be indicative rather than true. Despite the limitations mentioned above, we believe that this will most likely not affect our overall conclusion.

## 5. Conclusions

Reindeer meat contained higher vitamin B12, Fe, Zn and Se concentrations when compared to Norwegian beef, lamb, mutton, pork and chicken meat (two to twelve times higher depending on the nutrient). Thus, despite the low reindeer meat consumption in Norway compared to other meat types, the little amount consumed could significantly contribute to the recommended intake of such nutrients. The geographical differences revealed in this study were not large and will most likely have no impact for consumers. Vitamin E and Selenium demonstrated relatively large geographical variations, although no geographical gradient was observed for any of the studied nutrients. Sex had no significant effect in any of the essential elements, and no significant age effect was observed on Ca, Fe, Cr and Co concentrations. Calves had a significantly lower Zn concentration than young and older animals, whereas young animals had a significantly lower Se concentration than calves and older animals. Iron was positively correlated with calcium and vitamin B12 was positively correlated with zinc. Animals originating from districts with low animal population density had on average higher selenium concentration than those from districts with medium and high densities. There is a need for data on the quality of grazing districts, lichens distribution and summer flies intensity across the different grazing districts in order to get a better explanation for the observed geographical variations.
